# Dexamethasone Attenuates VEGF Expression and Inflammation but Not Barrier Dysfunction in a Murine Model of Ventilator–Induced Lung Injury

**DOI:** 10.1371/journal.pone.0057374

**Published:** 2013-02-25

**Authors:** Maria A. Hegeman, Marije P. Hennus, Pieter M. Cobelens, Annemieke Kavelaars, Nicolaas J. G. Jansen, Marcus J. Schultz, Adrianus J. van Vught, Cobi J. Heijnen

**Affiliations:** 1 Laboratory of Experimental Intensive Care and Anesthesiology, Academic Medical Center, Amsterdam, The Netherlands; 2 Laboratory of Neuroimmunology and Developmental Origins of Disease, University Medical Center Utrecht, Utrecht, The Netherlands; 3 Department of Pediatric Intensive Care, University Medical Center Utrecht, Utrecht, The Netherlands; 4 Department of Intensive Care Medicine, University Medical Center Utrecht, Utrecht, The Netherlands; 5 Department of Intensive Care, Academic Medical Center, Amsterdam, The Netherlands; University of Alabama-Birmingham, United States of America

## Abstract

**Background:**

Ventilator–induced lung injury (VILI) is characterized by vascular leakage and inflammatory responses eventually leading to pulmonary dysfunction. Vascular endothelial growth factor (VEGF) has been proposed to be involved in the pathogenesis of VILI. This study examines the inhibitory effect of dexamethasone on VEGF expression, inflammation and alveolar–capillary barrier dysfunction in an established murine model of VILI.

**Methods:**

Healthy male C57Bl/6 mice were anesthetized, tracheotomized and mechanically ventilated for 5 hours with an inspiratory pressure of 10 cmH_2_O (“lower” tidal volumes of ∼7.5 ml/kg; LV_T_) or 18 cmH_2_O (“higher” tidal volumes of ∼15 ml/kg; HV_T_). Dexamethasone was intravenously administered at the initiation of HV_T_–ventilation. Non–ventilated mice served as controls. Study endpoints included VEGF and inflammatory mediator expression in lung tissue, neutrophil and protein levels in bronchoalveolar lavage fluid, PaO_2_ to FiO_2_ ratios and lung wet to dry ratios.

**Results:**

Particularly HV_T_–ventilation led to alveolar–capillary barrier dysfunction as reflected by reduced PaO_2_ to FiO_2_ ratios, elevated alveolar protein levels and increased lung wet to dry ratios. Moreover, VILI was associated with enhanced VEGF production, inflammatory mediator expression and neutrophil infiltration. Dexamethasone treatment inhibited VEGF and pro–inflammatory response in lungs of HV_T_–ventilated mice, without improving alveolar–capillary permeability, gas exchange and pulmonary edema formation.

**Conclusions:**

Dexamethasone treatment completely abolishes ventilator–induced VEGF expression and inflammation. However, dexamethasone does not protect against alveolar–capillary barrier dysfunction in an established murine model of VILI.

## Introduction

Mechanical ventilation (MV) has the potential to cause progressive damage to pulmonary tissue, a phenomenon often referred to as ventilator–induced lung injury (VILI) [1], [2]. Non–physiologic stretch of lung cells may initiate disruption of alveolar–capillary barriers, eventually resulting in pulmonary dysfunction [3]–[5]. Vascular endothelial growth factor (VEGF) is recognized to be involved in the regulation of vascular permeability [6] and enhanced expression has been associated with vascular leakage in various experimental models of lung injury, including VILI [7]–[9]. Recently, VEGF short interfering (si) RNA was shown to attenuate pulmonary edema suggesting VEGF to critically regulate stretch–induced lung injury [7].

MV provokes a pro–inflammatory state of the lung, characterized by infiltration of neutrophils and release of inflammatory mediators [10]–[12]. Activated neutrophils can cause oxidative stress and protease activity in the alveoli, subsequently inducing severe disruption of pulmonary epithelial–endothelial barriers and leading to impaired gas exchange [13]. The awareness that neutrophils and inflammatory mediators are involved in the pathogenesis of severe inflammatory disorders, like the acute respiratory distress syndrome (ARDS), has led to the application of anti–inflammatory agents such as synthetic glucocorticoids [14], [15].

Glucocorticoids are a class of steroid hormones that bind to intracellular glucocorticoid receptors (GRs). In turn, the GR complex migrates to the nucleus where it inhibits nuclear factor (NF)–κB and activator protein (AP)–1 driven gene expression [16]. Moreover, glucocorticoids may suppress granulocyte activation and recruitment, preserve endothelial cell integrity and control vascular permeability [17]. Even though glucocorticoid therapy showed promising results on attenuating VILI in previous experimental models [18]–[20], treatment with glucocorticoids for ARDS is still under debate [21]–[23].

The present study was designed to examine the inhibitory effect of dexamethasone on VEGF expression, inflammation and alveolar–capillary barrier dysfunction in an established murine model of VILI. We applied a mild model of VILI using 5 hours of MV with clinically relevant ventilator settings, thereby preventing shock, metabolic acidosis and substantial damage to pulmonary architecture [24]. We hypothesized that down–regulation of VEGF expression by dexamethasone treatment would protect mice against important hallmarks of VILI, including inflammation, alveolar–capillary permeability, impaired gas exchange and pulmonary edema.

## Materials and Methods

### Animals

The animal care and use committees of the University Medical Center Utrecht and Academic Medical Center Amsterdam, the Netherlands, approved all experiments (permit number: DAA 100952). Animal handling was in accordance with institutional standards for care and use of laboratory animals. Eighty–eight healthy, male C57Bl/6 mice (20–24 grams; Charles River, Maastricht, the Netherlands) were randomly assigned to different experimental groups. Sixty–eight mice were randomized to MV, twenty mice to non–ventilated controls (NVC).

### Animal Handling

Animals were handled as described previously [25]. Mice received an intraperitoneal bolus of 1 ml sterile 0.9% saline. After 1 hour, mice were randomized to MV or NVC. Mice that were randomized to MV received an induction of anesthesia via intraperitoneal injection of a mix containing 126 mg/kg ketamine (Eurovet Animal Health BV, Bladel, the Netherlands), 0.1 mg/kg dexmedetomidine (Pfizer Animal Health BV, Capelle a/d IJssel, the Netherlands) and 0.5 mg/kg atropine (Pharmachemie, Haarlem, the Netherlands). Maintenance anesthesia was administered via an intraperitoneal cathether every hour and consisted of 36 mg/kg ketamine, 0.02 mg/kg dexmedetomidine and 0.075 mg/kg atropine. Sodium bicarbonate (200 mmol/l NaHCO_3_) was administered via the catheter every 30 minutes to compensate for metabolic acidosis. Body temperature was kept between 36.5 and 37.5°C.

### Mechanical Ventilation

After insertion of a Y–tube connector (1.0 mm outer diameter and 0.6 mm inner diameter; VBM Medizintechnik GmbH, Sulz am Neckar, Germany), mice were connected to a Servo Ventilator 900C (Siemens–Elema, Solna, Sweden) and mechanically ventilated for 5 hours in a pressure–controlled mode, at a fractional inspired oxygen concentration (FiO_2_) of 0.5, inspiration to expiration ratio of 1∶1 and positive end–expiratory pressure of 2 cmH_2_O. MV was initiated with an inspiratory pressure of 10 cmH_2_O (“lower” tidal volumes of ∼7.5 ml/kg; LV_T_) or 18 cmH_2_O (“higher” tidal volumes of ∼15 ml/kg; HV_T_). Respiratory rate was set at 100 and 50 breaths/minute, respectively, aiming at normo–pH (7.35–7.45). A sigh of 30 cmH_2_O for five breaths was performed every 30 minutes, aiming at normo–PaCO_2_ (35–45 mmHg).

### Dexamethasone Treatment

Dexamethasone (20 µg per animal; Bufa, Hilversum, the Netherlands) was intravenously administered at initiation of HV_T_–ventilation. Control mice received the same volume of sterile 0.9% saline (vehicle) intravenously.

### Monitoring

Systolic blood pressure and heart rate were non–invasively monitored using a tail–cuff system for mice (ADInstruments, Spenbach, Germany). After 5 hours of MV, arterial blood was taken from the carotid artery for blood gas analysis (Rapidlab 865; Bayer, Mijdrecht, the Netherlands).

### Wet to Dry Ratio

The left lung was weighed, dried for three days in a 65°C stove and weighed again.

### Bronchoalveolar Lavage

The right lung was lavaged by instilling 3×0.5 ml sterile saline into the trachea. Total cell counts were determined in bronchoalveolar lavage fluid (BALF) using a hemacytometer (Beckman Coulter, Fullerton, CA). Differential counts were performed on BALF cytospin preparations stained with Giemsa (Diff–Quick; Dade Behring AG, Düdingen, Switzerland). Cell–free supernatant was used to measure total protein (BCA protein assay; Pierce Biotechnology, Rockford, IL).

### Histopathology

The left lung of a second subset of animals was filled with Tissue–Tek (Sakura Finetek, Zoeterwoude, the Netherlands), snap frozen and cut to 5 µm cryosections. Longitudinal sections were stained with hematoxylin–eosin (H&E; Klinipath, Duiven, the Netherlands).

### Homogenates

The right lung of a second subset of animals was pulverized using a liquid nitrogen–cooled mortar/pestle and divided in several fractions allowing us to perform multiple analyses.

### Real–time RT–PCR

Total RNA was isolated with TRIzol® (Invitrogen, Paisley, UK). cDNA was synthesized with SuperScript Reverse Transcriptase (Invitrogen). PCR reaction was performed with iQ5 Real-Time PCR Detection System (Bio–Rad Laboratories, Hercules, CA) using primers for keratinocyte–derived chemokine (KC); for primer sequences see [26]. PCR product size was verified on gel to confirm appropriate amplification. Data were normalized for expression of internal controls, i.e. the average value of β–actin and glyceraldehyde 3–phosphate dehydrogenase (GAPDH).

### Multiplex Cytokine Analysis

125 µg protein was analyzed for KC, macrophage inflammatory protein (MIP)–2, monocyte chemotactic protein (MCP)–1, interleukin (IL)–1β, IL–6, IL–10 and vascular endothelial growth factor (VEGF) by multiplex cytokine assay using a Luminex analyzer (Bio–Rad) according to manufacturer’s instructions (R&D systems, Minneapolis, MN).

### Statistical Analysis

Data are expressed as median (IQR) or boxplot (min–max). Arterial blood gas variables were analyzed by Mann Whitney tests (vehicle versus dexamethasone). As group characteristics did not follow a normal distribution, all other parameters were analyzed by Kruskal Wallis tests with post–hoc Mann Whitney tests and Bonferroni correction. First, we compared HV_T_ with LV_T_, HV_T_ with NVC and LV_T_ with NVC (p–value for significance was set at 0.0167). Next, we compared HV_T_ vehicle with HV_T_ dexamethasone and HV_T_ vehicle with NVC (p–value for significance was set at 0.025). Outliers, defined as >2SD, were excluded from analysis.

## Results

### Ventilator–induced Lung Injury

**Figure 1:**
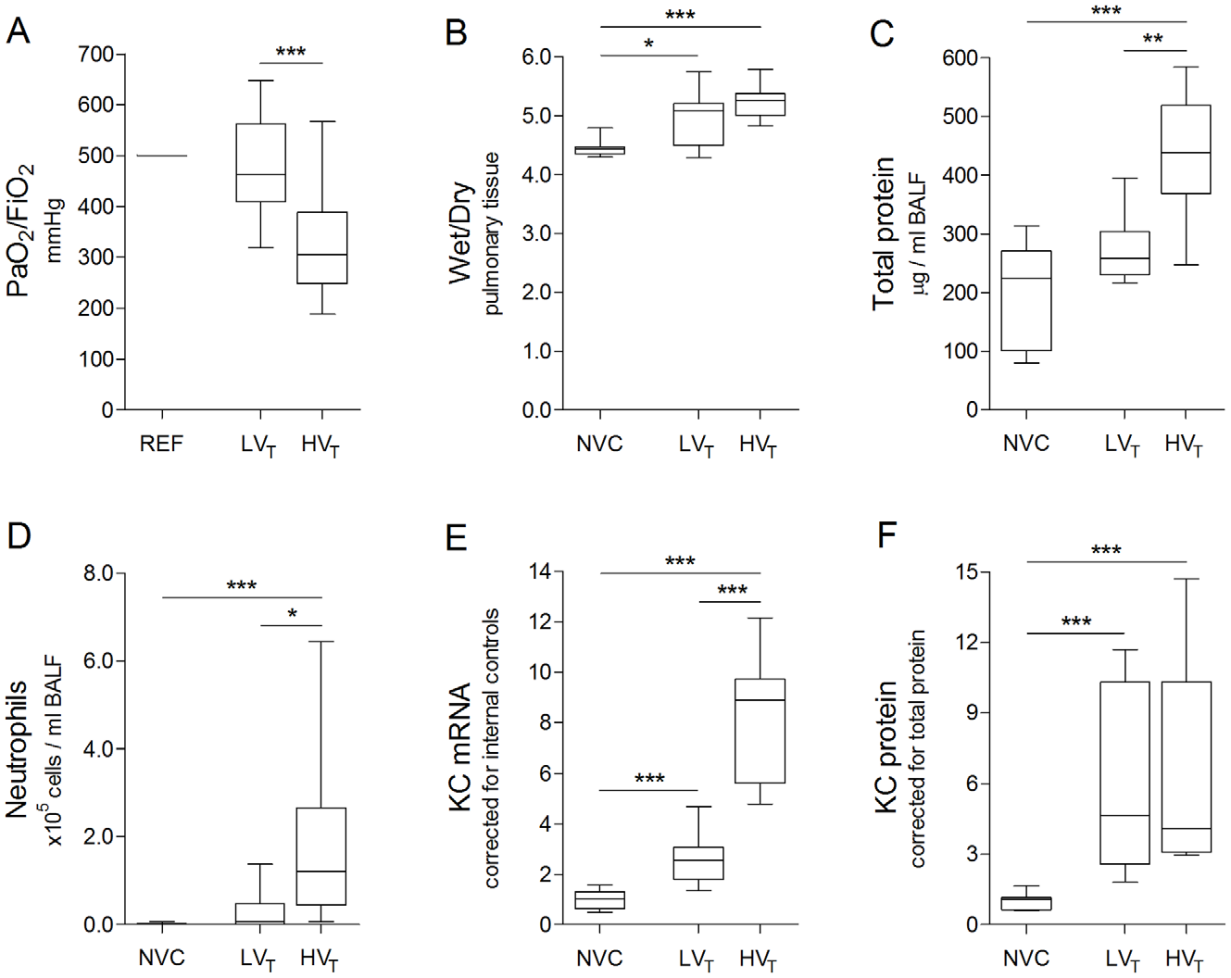
Ventilator–induced lung injury. **A:** Pulmonary function is represented by the ratio of arterial oxygen partial pressure to fractional inspired oxygen concentration (PaO_2_/FiO_2_). **B:** Pulmonary edema is represented by wet to dry ratio of lung tissue (Wet/Dry). **C:** Alveolar–capillary permeability is represented by total protein levels in bronchoalveolar lavage fluid (BALF). **D:** On BALF cytospin preparations, differential cell counts were performed to determine neutrophil exudation into the alveolar space. **E:** In total lung homogenates, mRNA expression of the chemo–attractant keratinocyte–derived chemokine (KC) was determined by real–time RT–PCR. **F:** In total lung homogenates, protein expression of KC was determined by multiplex cytokine analysis. Expression levels were normalized for internal control concentrations (E), i.e. the average value of β–actin and glyceraldehyde 3–phosphate dehydrogenase (GAPDH), or total protein concentrations (F). Data are expressed as boxplot (min–max) of 18–22 (A) or 6–16 (B–F) mice per group (* p<0.05, ** p<0.01, *** p<0.001). E–F: Data are depicted relative to non–ventilated controls (NVC). REF = assumed PaO_2_/FiO_2_ ratio from mice with non–injured lungs; LV_T_, HV_T_ = mechanically ventilated with low or high tidal volumes.

All mice survived the experimental procedures and were sacrificed thereafter. Healthy mice developed signs of VILI when exposed to MV for 5 hours using clinically relevant ventilator settings. PaO_2_ to FiO_2_ ratios were lower whereas BALF protein levels and BALF neutrophil counts were higher in HV_T_–ventilated mice, but not in LVT–ventilated mice (figure 1a,c,d). Pulmonary wet to dry ratios, KC mRNA and protein levels were increased in both LV_T_ and HV_T_–ventilated mice (figure 1b,e,f). Compared with LV_T_–ventilation, HV_T_–ventilation was associated with lower PaO_2_ to FiO_2_ ratios, higher BALF total protein levels, higher BALF neutrophil counts and more pulmonary KC mRNA levels (figure 1a,c–e).

### Dexamethasone does not Affect Hemodynamic and Arterial Blood Gas Variables

Since VILI was most pronounced after HV_T_–ventilation, we continued the investigations by focusing on this specific group. Heart rates and systolic blood pressures remained stable for the complete duration of HV_T_–ventilation (table 1a). Carbon dioxide tension (PaCO_2_), pH and base excess (BE) maintained within the normal to near–normal range (table 1b). Mice were intravenously treated with sterile saline (vehicle) or dexamethasone at the initiation of HV_T_–ventilation and subsequently ventilated for 5 hours. No significant changes on hemodynamic conditions and blood gas variables were observed after dexamethasone treatment (table 1).

**Table 1:** Hemodynamic and arterial blood gas variables.

			Veh	Dex
A	HR	t = 0 h	392.5 [315.0–408.8]	397.5 [381.3–410.0]
		t = 2.5 h	352.5 [287.5–377.5]	360.0 [295.0–378.8]
		t = 5 h	365.0 [350.0–385.0]	342.5 [292.5–392.5]
	BP	t = 0 h	97.5 [81.3–108.8]	112.5 [88.8–137.5]
		t = 2.5 h	70.0 [61.3–83.8]	75.0 [60.0–90.0]
		t = 5 h	72.5 [61.3–82.5]	80.0 [61.3–93.8]
B	PaO_2_	t = 5 h	152.7 [129.5–191.7]	164.1 [133.0–206.1]
	PaCO_2_	t = 5 h	36.0 [28.3–39.3]	38.6 [32.7–41.8]
	pH	t = 5 h	7.51 [7.45–7.57]	7.46 [7.42–7.56]
	BE	t = 5 h	4.15 [2.50–7.05]	3.10 [1.50–6.25]

Veh, Dex = mechanically ventilated and treated with vehicle or dexamethasone; HR = heart rate in beats per minute; BP = systolic blood pressure in mmHg; PaO_2_ = partial pressure of arterial oxygen in mmHg; PaCO_2_ = partial pressure of arterial carbon dioxide in mmHg; BE = base excess in mmol/l. Data are presented as median [IQR] of 6–8 (A) or 10–16 (B) mice per group.

### Dexamethasone Inhibits Ventilator–induced VEGF Expression

**Figure 2:**
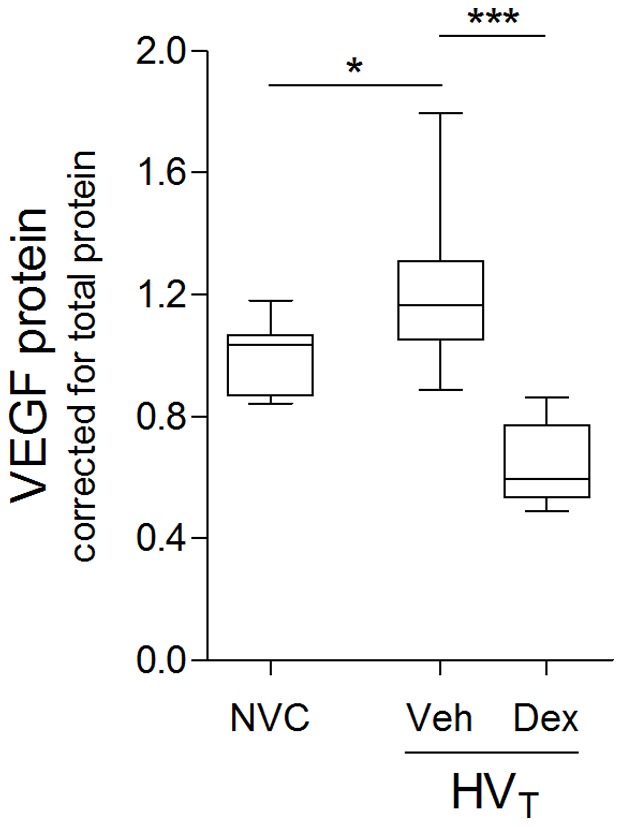
Vascular endothelial growth factor (VEGF). In total lung homogenates, protein expression of VEGF was determined by multiplex cytokine analysis. Levels were normalized for total protein concentrations. Data are expressed as boxplot (min–max) of 6–10 mice per group (* p<0.05, *** p<0.001). Data are depicted relative to non–ventilated controls (NVC). Veh, Dex = intravenously treated with either vehicle (sterile saline) or dexamethasone; HV_T_ = mechanically ventilated with high tidal volumes.

We examined the inhibitory effect of dexamethasone on VEGF expression by measuring protein levels in total lung homogenates. Dexamethasone treatment significantly inhibited ventilator–induced VEGF expression when compared to vehicle treatment, even below basal level (p<0.01, HV_T_ Dex versus NVC) (figure 2).

### Dexamethasone Inhibits Ventilator–induced Inflammatory Response

**Figure 3:**
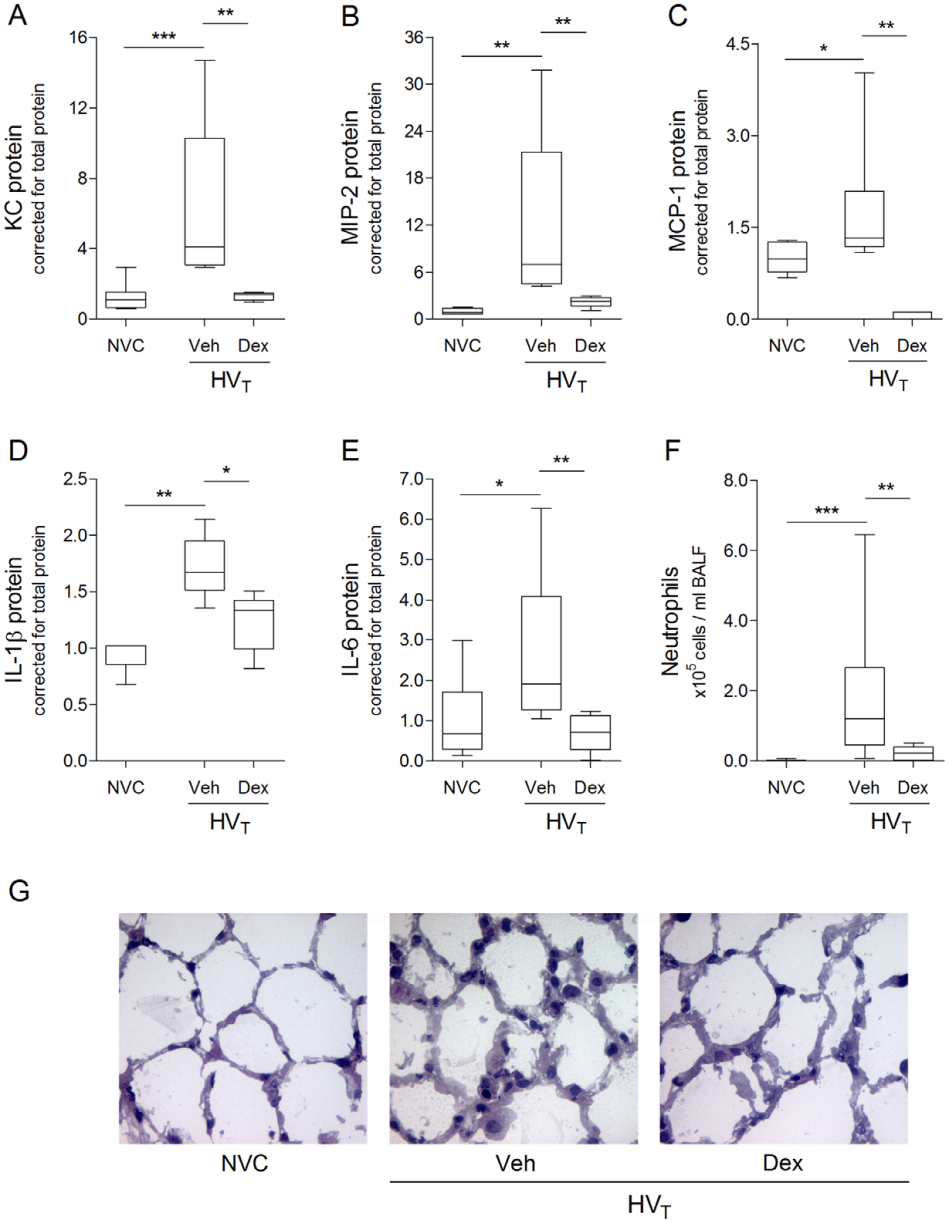
Pro–inflammatory response. **A–E:** In total lung homogenates, protein expression of the chemo–attractants keratinocyte–derived chemokine (KC), macrophage inflammatory protein (MIP)–2, monocyte chemotactic protein (MCP)–1 and pro–inflammatory cytokines interleukin (IL)–1β, IL–6 was determined by multiplex cytokine analysis. Levels were normalized for total protein concentrations. **F:** On bronchoalveolar lavage fluid (BALF) cytospin preparations, differential cell counts were performed to determine neutrophil exudation into the alveolar space. Data are expressed as boxplot (min–max) of 5–10 (A–E) or 8–16 (F) animals per group (* p<0.05, ** p<0.01, *** p<0.001). A–E: Data are depicted relative to non–ventilated controls (NVC). **G:** Pulmonary sections were stained with hematoxylin–eosin (H&E) to analyze lung histology and presence of granulocytes in lung tissue. Magnification ×500. Veh, Dex = intravenously treated with either vehicle (sterile saline) or dexamethasone; HV_T_ = mechanically ventilated with high tidal volumes.

To investigate whether an anti–inflammatory action of dexamethasone was detectable in the murine model of VILI, we determined protein expression of inflammatory mediators in total lung homogenates. Dexamethasone inhibited ventilator–induced protein expression of the chemokines KC, MIP–2 and MCP–1 (figure 3a–c) and the pro–inflammatory cytokines IL–1β and IL–6 (figure 3d–e). The anti–inflammatory cytokine IL–10 was below detection level in all experimental groups. In addition, ventilator–induced granulocyte infiltration was significantly attenuated after dexamethasone treatment as shown by diminished neutrophil numbers on BALF cytospin preparations (figure 3f). We stained lung sections for H&E to visualize the effects of dexamethasone on histology. Figure 3g shows 5 hours of HV_T_–ventilation to cause thickening of alveolar walls and granulocyte margination to blood vessel walls. Confirming the quantitative measure for granulocyte infiltration, dexamethasone treatment diminished ventilator*–*induced granulocyte margination compared to vehicle treatment (figure 3g).

### Dexamethasone Fails to Improve Alveolar–capillary Barrier Dysfunction

**Figure 4:**
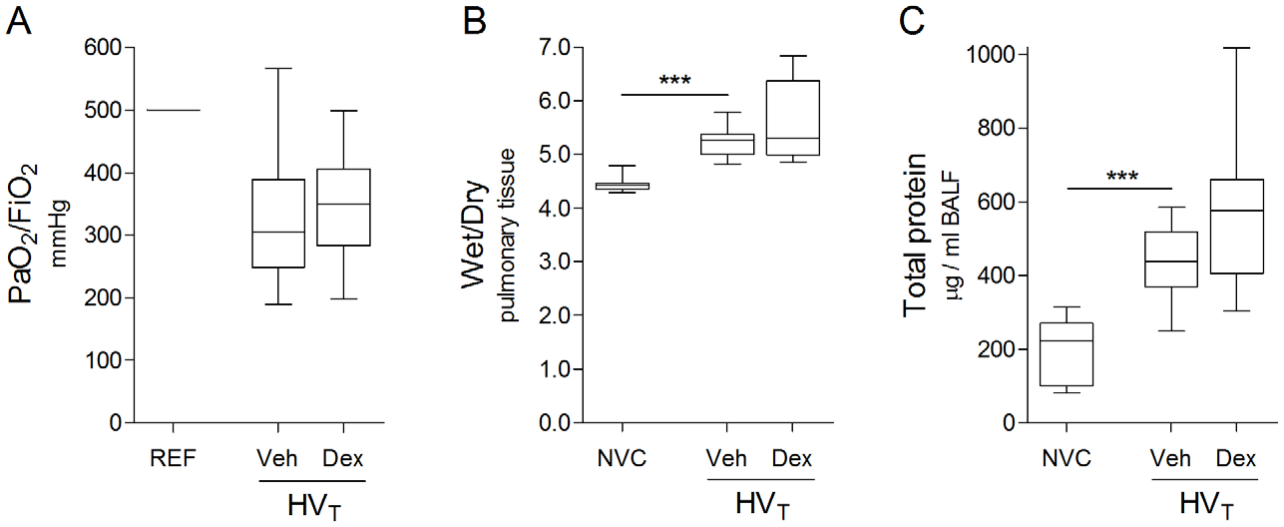
Alveolar–capillary barrier dysfunction. **A:** Pulmonary function is represented by the ratio of arterial oxygen partial pressure to fractional inspired oxygen concentration (PaO_2_/FiO_2_). **B:** Pulmonary edema is represented by wet to dry ratio of lung tissue (Wet/Dry). **C:** Alveolar–capillary permeability is represented by total protein levels in bronchoalveolar lavage fluid (BALF). Data are expressed as boxplot (min–max) of 11–18 (A) or 9–12 (B–C) mice per group (*** p<0.001). REF = assumed PaO_2_/FiO_2_ ratio from mice with non–injured lungs; NVC = non–ventilated controls (NVC); Veh, Dex = intravenously treated with either vehicle (sterile saline) or dexamethasone; HV_T_ = mechanically ventilated with high tidal volumes.

Since enhanced VEGF expression and pro–inflammation may initiate alveolar–capillary barrier dysfunction, we evaluated whether dexamethasone administration at the initiation of HV_T_–ventilation would protect against alveolar–capillary barrier dysfunction. Dexamethasone treatment failed to restore impaired gas exchange induced by 5 hours of HV_T_–ventilation, as represented by comparable PaO_2_ to FiO_2_ ratios of mice treated with dexamethasone and those treated with vehicle (figure 4a). In addition, no protective effects of dexamethasone were observed on pulmonary wet to dry ratios and BALF total protein levels (figure 4b,c). Dexamethasone even seemed to worsen ventilator–induced effects on these measures of vascular leakage, although differences did not reach statistical significance.

## Discussion

The present study shows that dexamethasone treatment prevents VEGF expression in lungs of 5 hour HV_T_–ventilated mice. Moreover, dexamethasone markedly inhibits neutrophil influx and inflammatory mediator expression. However, dexamethasone fails to protect against alveolar–capillary barrier dysfunction in this model, since BALF protein levels, lung wet to dry ratios and PaO_2_ to FiO_2_ ratios were not affected by dexamethasone treatment.

MV has the potential to cause alveolar–capillary barrier dysfunction. Experimental studies demonstrated that non–physiologic stretch of lung tissue disrupts alveolar–capillary barriers, eventually leading to impaired pulmonary function [3]–[5]. It has been recognized that VEGF plays a crucial role in the structural maintenance of mature lung tissue [27]. Although both deleterious and protective effects of VEGF have been proposed in pathologic lung conditions [28], elevated VEGF levels have been related to vascular leakage in experimental models of VILI [7], [8]. Moreover, recently a critical involvement of VEGF was proposed in the regulation of stretch–induced lung injury [7]. The clinically relevant ventilator settings used in current VILI model stimulated VEGF production in lungs of healthy mice, which was accompanied by microvascular permeability, pulmonary edema formation and impaired gas exchange. These data suggest VEGF to be associated with alveolar–capillary barrier dysfunction in the pathogenesis of VILI. Since the inhibitory effect of glucocorticoids on VEGF–induced vascular leakage was shown in other experimental models [29]–[31], we evaluated whether dexamethasone also prevented VEGF expression induced by 5 hours of HV_T_–ventilation. Dexamethasone treatment at the initiation of HV_T_–ventilation caused a significant down–regulation of VEGF expression in lungs of ventilated mice, even below basal level, which may positively affect the development of VILI.

Besides enhanced VEGF expression, activated granulocytes may also damage alveolar–capillary barriers thereby impairing pulmonary function [13]. The importance of granulocyte–mediated tissue injury in VILI has been described previously [10]. Indeed, depletion of granulocytes showed to improve gas exchange, vascular leakage and hyaline membrane formation in rabbits exposed to repetitive lung lavage and high–frequency oscillatory ventilation [10]. Present investigation reveals that dexamethasone treatment at the initiation of HV_T_–ventilation prevents neutrophil infiltration into lungs of ventilated mice, consistent with previous findings [19], [32]. In addition, dexamethasone completely abolished inflammatory mediator expression confirming that this synthetic glucocorticoid is capable of preventing lung inflammation induced by MV. It may well be that down–regulation of VEGF expression contributes to the anti–inflammatory effect of dexamethasone. Evidence for this suggestion has been provided by a recent observation that knock–down of VEGF by siRNA diminishes ventilator–induced neutrophil sequestration [7].

Even though dexamethasone treatment significantly inhibited VEGF expression and pro–inflammation, it failed to restore alveolar–capillary barrier dysfunction in a VILI model using clinically relevant ventilator settings. The finding that dexamethasone does not have an impact on lung function supports recent experimental data [33]. However, these results are in apparent contrast with earlier research describing the protective effects of glucocorticoid treatment on lung injury [18]–[20]. In this regard, methylprednisolone therapy in rats caused a leftward shifting of the pressure–volume (P–V) curve in rats ventilated for 40 minutes [19]. Yet, deterioration of the P–V curve was still clear despite reduced granulocyte numbers. As mechanical stretch may contribute to lung injury to a great extent, investigators proposed that it may be difficult to completely inhibit the stretch–induced effects on lung injury merely by anti–inflammatory therapy [19]. As mice in the present study were mechanically ventilated for 5 hours, it may well be that alveolar–capillary barrier dysfunction induced by longer periods of mechanical stretch are not affected by anti–inflammatory actions of dexamethasone.

While the use of glucocorticoids has shown promising results on attenuating lung injury in previous experimental models of VILI [18]–[20], the efficacy of glucocorticoids in treating ARDS in critically ill patients is still under debate [21]–[23]. Clinically, there is some support for our current experimental data. Indeed, a randomized controlled trial described that more ventilated patients developed ARDS after methylprednisolone treatment when compared to placebo [34]. Enhanced incidence of ARDS was also described in another clinical trial where glucocorticoid therapy was started within 2 hours after the onset of sepsis [35]. Furthermore, these authors demonstrated that the 14–day mortality rate was significantly higher in patients treated with methylprednisolone [35]. So, these previous randomized controlled trials failed to show protective effects of early, high dose corticosteroid treatment in patients at risk for ARDS. In line with these clinical observations, we observed that dexamethasone treatment in mice without pre–existing lung injury did not restore alveolar–capillary barrier dysfunction induced by MV. We cannot exclude, however, that multiple injections of dexamethasone would show some protective effects on lung injury as more recent clinical trails revealed that prolonged treatment with moderate doses of corticosteroids may improve clinical outcome of ARDS patients [36], [37].

Intriguingly, alveolar–capillary permeability and pulmonary edema (i.e. vascular leakage) tended to deteriorate in HV_T_–ventilated mice treated with dexamethasone. One explanation for these findings may be that dexamethasone reduces VEGF expression even below basal level, as shown in our present study. Although increasing evidence suggests a harmful role for exaggerated VEGF expression in the pathogenesis of VILI [7], protective effects of VEGF are proposed in pathologic lung conditions as well [28]. For instance, VEGF has been shown to function as a survival factor for epithelial and endothelial cells [7], [38], [39]. Significant inhibition of VEGF expression may therefore withhold recovery of lung injury. In line with this notion, the anti–inflammatory action of dexamethasone may attenuate the growth–promoting effects of inflammatory mediators thereby suppressing potential repair mechanisms [40]. Another explanation may be that mice were exposed to MV with relatively high oxygen levels (FiO_2_ of 0.5, moderate hyperoxia). It has been reported that dexamethasone increased extravascular lung water in hyperoxia–exposed rats causing a shift in the onset of hyperoxic lung injury to an earlier time point [41]. The moderate hyperoxia used in our MV strategy may therefore counterbalance the positive effects of dexamethasone on pulmonary injury so that the overall effect is neutral or even deleterious.

Taken together, the present study clearly demonstrated that dexamethasone treatment is capable of attenuating important aspects of VILI. However, inhibition of VEGF expression and pro–inflammation by dexamethasone does not necessarily improve alveolar–capillary barrier function as well. In line, we previously described that the vessel protective factor angiopoietin (Ang)–1 only protects against ventilator–induced VEGF expression and pro–inflammation, not against alveolar–capillary barrier dysfunction [25]. The other way around, pharmacological agents that attenuate alveolar–capillary barrier dysfunction due to MV may not influence inflammation [42], [43]. The assumption that lung inflammation and injury are occurring sequentially in the pathogenesis of VILI may therefore be questioned. Indeed, dissociation of inflammatory mediators and physiologic function has been described recently in experimental models of lung injury [33].

It should be noted that healthy mice were used in present study. So, it may well be that anti–inflammatory agents are still beneficial in subjects with pre–existing lung inflammation. The fact that glucocorticoid therapy showed protective effects when started in patients with established ARDS [44], [45], supports this hypothesis. Additional evidence was provided by a retrospective analysis of a clinical trial studying the effects of methylprednisolone treatment in view of the precipitating cause of ARDS (for instance infectious or non–infectious) [46]. The findings of this analysis suggest that the underlying mechanisms and thus the response to anti–inflammatory treatment may differ with the cause of lung injury [46].

### Conclusions

Dexamethasone treatment completely abolishes the VEGF expression and pro–inflammation induced by 5 hours of HV_T_–ventilation. However, it fails to protect against alveolar–capillary barrier dysfunction. On the basis of our current findings, we propose that lung injury induced by longer periods of mechanical stretch may not be responsive to dexamethasone treatment.
